# Differential unfolded protein response during Chikungunya and Sindbis virus infection: CHIKV nsP4 suppresses eIF2α phosphorylation

**DOI:** 10.1186/1743-422X-10-36

**Published:** 2013-01-28

**Authors:** Abhay P S Rathore, Mah-Lee Ng, Subhash G Vasudevan

**Affiliations:** 1 Program in Emerging Infectious Diseases, Duke-NUS Graduate Medical School, 8-College Road, Singapore, 169857, Singapore; 2 Department of Microbiology, National University of Singapore, Singapore, 117597, Singapore

## Abstract

Chikungunya (CHIKV) and Sindbis (SINV) are arboviruses belonging to the alphavirus genus within the Togaviridae family. They cause frequent epidemics of febrile illness and long-term arthralgic sequelae that affect millions of people each year. Both viruses replicate prodigiously in infected patients and *in vitro* in mammalian cells, suggesting some level of control over the host cellular translational machinery that senses and appropriately directs the cell’s fate through the unfolded protein response (UPR). The mammalian UPR involves BIP (or GRP78), the master sensor in the endoplasmic reticulum (ER) together with the three downstream effector branches: inositol-requiring ser/thr protein kinase/endonuclease (IRE-1), PKR-like ER resident kinase (PERK) and activating transcription factor 6 (ATF-6). Through careful analysis of CHIKV and SINV infections in cell culture we found that the former selectively activates ATF-6 and IRE-1 branches of UPR and suppresses the PERK pathway. By separately expressing each of the CHIKV proteins as GFP-fusion proteins, we found that non-structural protein 4 (nsP4), which is a RNA-dependent-RNA polymerase, suppresses the serine-51 phosphorylation of eukaryotic translation initiation factor, alpha subunit (eIF2α), which in turn regulates the PERK pathway. This study provides insight into a mechanism by which CHIKV replication responds to overcome the host UPR machinery.

## Introduction

Chikungunya virus (CHIKV) is a member of the alphavirus genus, which contains 26 known arboviruses with a wide host range [1]. During the past 50 years, numerous CHIKV epidemics have been documented in both Africa and Asia [2]. Since, its discovery, CHIKV has spread widely and currently Chikungunya fever has been detected in nearly 40 countries with a potential to affect millions of people worldwide [3]. In general, alphaviruses are divided into viruses that cause human diseases characterized by rash and arthritis, that are primarily found in the “old world” such as CHIKV, O nyong nyong, Sindbis (SINV), Ross River, Barmah Forest and Mayaro virus [4] and viruses that cause encephalitis, which are primarily found in the “new world”. The first clear association of an alphavirus with arthritic disease was made in 1953 when CHIKV was isolated from the blood of individuals in Tanzania with severe arthritis [5]. SINV was first isolated in 1952, which causes similar disease to CHIKV in humans known as sindbis fever and the symptoms include arthralgia, rash and malaise [6]. These arthritogenic alphaviruses share certain antigenic determinants [4] and also considerable genome similarity that makes them interesting for comparative responses to the host. In humans, CHIKV infection is characterized by a rapid onset of fever that is cleared in 5–7 days with long lasting immunity [7]. The major pathology associated with CHIKV infection is very high viremia and polyarthritis [8-11]. The mortality rate associated with CHIKV infection has been estimated to be 1:1000 with most deaths occurring in neonates, adults with underlying conditions and the elderly [3]. The persistent detection of viral RNA or antigen in the host has suggested the long-term persistence of these viruses in humans [12,13]. The alphavirus genome is a single-stranded RNA genome of ~12 kb in size of positive polarity. It encodes two polyproteins of which the first encodes nonstructural proteins (nsPs) 1–4: nsP1 contains methyl transferase and guanyl transferase activities, nsP2 is a helicase/protease, nsP3 is an accessory protein involved in RNA synthesis and nsP4 is the RNA dependent RNA polymerase. The second polypeptide, translated from a subgenomic RNA codes for structural proteins, capsid (C) and the envelope glycoproteins, E1 and E2 that constitute the virion coat [4,14,15]. Several studies have shown that alphavirus replication in mammalian cells usually results in severe cytopathicity, mainly caused by dramatic shutdown of host translation machinery [16-20]. However, the mechanism by which CHIKV maintains such a high replication rate in the infected cells is poorly understood.

One host response mechanism that has the potential to limit virus replication is the endoplasmic reticulum (ER) stress response, also known as unfolded protein response (UPR) which, maintains cellular protein homeostasis and prevents the over-accumulation of unfolded proteins in the lumen of the ER during normal and diseased states [21]. ER chaperone immunoglobulin heavy chain binding protein (BIP), also known as glucose regulated protein 78 (GRP78) plays a central role in this process via a three-pronged regulatory pathway involving PKR-like ER kinases (PERK), activating transcription factor 6 (ATF-6) and the ER transmembrane protein kinase/endoribonuclease (IRE-1). Under stress conditions, BIP is sequestered to misfolded or unfolded proteins in the ER whereupon it activates PERK, ATF-6 and IRE-1 [22]. During UPR, PERK activates by self-dimerization and phosphorylation. Activated PERK phosphorylates eIF2α at serine-51 and leads to an inhibition of general protein synthesis. PERK activation also induces the activation of C/EBP homologous protein (CHOP) and growth arrest and DNA damage-inducible protein GADD34 [23]. CHOP is responsible for apoptosis mediated cell death when functions of ER are severely impaired to protect the organism by eliminating the damaged cell [24] whilst GADD34 and its binding partner protein phosphatase-1 catalytic subunit (PP1c) are involved in eIF2α de-phosphorylation that also modulates cell fate during protein translational stress. The activation of IRE-1 branch of UPR pathway leads to transcription induction of a subset of genes encoding protein degradation and pro-survival enzymes such as components of ER associated degradation (ERAD) including ER degradation-enhancing-α-mannosidase like protein (EDEM) [25-27]. Autoproteolytic activation of ATF-6 stimulates transcription of genes encoding chaperones that assist in the refolding of misfolded proteins [28]. On balance, the UPR pathway in conjunction with ERAD controls the survival vs apoptosis decision of cells stressed by increased protein translation from external stimulus [29].

To circumvent the host cellular translational response, several viruses [respiratory syncytial virus, simian virus-5, Tula virus, African swine fever virus, herpes simplex virus, cytomegalovirus, dengue virus and hepatitis C virus [30-34] have been shown to regulate UPR machinery. For example, in the case of hepatitis C virus, the virus encoded NS5A phosphoprotein, inhibits PKR activation by direct protein-protein interaction [35]. Likewise, K3L gene product of vaccinia virus also binds to PERK and inhibits its activation [36]. Others such as herpes simplex viruses encode proteins that mimic host factors to regulate the protein synthesis traffic [37]. In light of these various mechanisms by which viruses modulate UPR pathway, we investigated the impact of CHIKV replication on the various components of the UPR machinery and compared it to another representative alphavirus, SINV, in order to reveal differential host responses to these unique but closely related pathogens. Real-time RT-PCR monitoring of transcriptional changes and Western blotting of infected cells were used to reveal the UPR components during both CHIKV and SINV infections. By carefully examining the UPR pathway components and by selectively inducing the ER stress using thapsigargin or tunicamycin treatment, we identified the suppression of eIF2α phosphorylation during CHIKV infection in the early phase of virus replication that does not occur with SINV infection. Subsequently, transfection of individual CHIKV-encoded proteins as GFP-fusion proteins revealed a mechanistic basis for the phenomenon dependent on nsP4.

## Materials and methods

### Cells and viruses

Mosquito cells *Aedes albopictus* clone (C6/36) and baby hamster kidney cells (BHK-21) were cultured in RPMI-1640 medium (Gibco) supplemented with 10% fetal bovine serum (FBS) (Gibco). Human embryonic kidney cells (HEK293) and human lung fibroblast cells (MRC-5) were cultured in DMEM (Gibco) supplemented with 10% FBS. C6/36 cells were grown and maintained in 28°C temperature incubator. BHK-21, MRC-5 and HEK293 cells were grown and maintained at 37°C in a humidified incubator with 5% CO_2_ atmosphere. CHIKV strain ‘ROSS’ and a laboratory strain of SINV MRM-39 strain (isolated in Australia [38]) was a generous gift from Dr. Ooi Eng Eong (Duke-NUS GMS). Both the viruses were amplified in C6/36 cells supplemented with 5% FBS at 28°C and titrated by plaque assay as described previously [39]. Low passage number (below passage 5) was used for performing all experiments. Tunicamycin (Sigma) or thapsigargin (sigma) was used to induce UPR stress in the cells.

### *In vitro* virus quantification

Prior to their use, plaque assays were carried out to quantify the number of infectious viral particles for CHIKV and SINV viruses used in the study. Briefly, BHK-21 cells were cultured to approximately 80% confluency in 24-well plates (NUNC). The virus stock was 10-fold serially diluted from 10^−1^ to 10^−12^ in RPMI 1640 (Gibco). BHK-21 monolayers were infected with 200μl of each virus dilution. After incubation at 37°C and 5% CO_2_ atmosphere for 1h with rocking at 15 min intervals, the medium was decanted and 1ml of 1% (w/v) carboxymethyl cellulose in RPMI supplemented with 2% FBS was added to each well. After 72h of incubation at 37°C in 5% CO_2_, the cells were fixed with 4% paraformaldehyde and stained for 30 min with 200 μl of 1% crystal violet dissolved in 1X-PBS. After thorough rinsing with water, the plates were dried and the plaques were scored visually.

### Primer sequences used in the study

***Real-time PCR primer sequences: -*** CHIKV nsP1 (F-TAGAGCAGGAAATTGATCCC, R- CTTTAATCGCCTGGTGGTAT), SINV E1 (F-CACCCCGCACAAAAATGAC, R- AAAAGGGCAAACAGCCAACTC), EDEM (F-TCATCCGAGTTCCAGAAAGCAGTC, R- TTGACATAGAGTGGAGGGTCTCCT), XBP-1 (F-TCACCCCTCCAGAACATCTC, R- ACTGGGTCCAAGTTGTCCAG), CHOP (F-TCTGATTGACCGAATGGTG, R- TCTGGGAAAGGTGGGTAGTG), BIP (F-TAGTGCAAGCTGAAGGCTGA, R- GGGCTGGAGTACAGTGGTGT), GADD34 (F-AACCTCTACTTCTGCCTTGTCT, R- CGCCTCTCCTGAACGATACTC), eIF2αK2 (F-TTTGGACAAAGCTTCCAACC, R- ACTCCCTGCTTCTGACGGTA), 18s (F-TGTTCAAAGCAGGCCCGAG, R-CGGAACTACGACGGTATCTGATC), GAPDH (F- ACAGTCAGCCGCATCTTCTT, R- ACGACCAAATCCGTTGACTC), Actin (F-CAGGGGAACCGCTCATTGCCAATGG, R-TCACCACACACTGTGCCCATCTACGA), XBP-1 splicing (F- AAACAGAGTAGCAGCTCAGACTGC, R- TCCTTCTGGGTAGACCTCTGGGAG).

***CHIKV recombination cloning primer sequences: -*** nsP1 (F- AGATCTCGAGCTCAAGCT TCG**ATGGATCCTGTGTACGTG**, R- TTAACCGTCGACTGCAGA**TCCTGCACCCGCTCTGTC**), nsP2 (F- TCCGGACTCAGATCTCGAGCT**ATAATAGAGACTCCGAGAGGA**, R-GGATCCCGGGCCCGCGGTACC**ACATCCTGCTCGGGTGGC**), nsP3 (F- TCCGGACTCAGATCTCGAGCT**GCACCGTCGTACCGGGTA**, R- GGATCCCGGGCCCGCGGTACC**CCCACCTGCCCTGTCTAG**), nsP4 (F- TCCGGACTCAGATCTCGAGCT**TATATATTCTCGTCGGAC**, R- GGATCCCGGGCCCGCGGTACC**CTATTTAGGACCGCCGTA**), Capsid (F- TCCGGACTCAGATCTCGAGCT**TGCATGAAAATCGAAAATGAC**, R- GGATCCCGGGCCCGCGGTACC**CCACTCTTCGGCTCCCTC**), E2 (F- AGATCTCGAGCTCAAGCTTCG**CCATACTTAGCTCACTGT**, R- TTATCTAGATCCGGTGGATCC**GCAGCATATTAGGCTAAG**), E1 (F- AGATCTCGAGCTCAAGCTTCG**AGAACAGCTAAAGCGGCC**, R- TTATCTAGATCCGGTGGATCC**TTAGTGCCTGCTGAACGA**).

### RNA extraction and real-time RT-PCR analysis

HEK293 cells (1×10^5^) were infected with virus (CHIKV/SINV) at a multiplicity of infection (MOI) of 1. At indicated time intervals, total RNA was isolated using the trizol (Invitrogen) extraction method and 1μg of total RNA was used for cDNA synthesis using ImProm II reverse transcription system (Promega), with oligo dT as primer. cDNA (50 ng) was used for real-time amplification of specific genes using respective primers (Materials and Methods) in Bio-Rad iQ-5 real time thermal cycler. The expression of viral and host gene products was normalized to Actin and GAPDH mRNA expression, followed by normalization to expression levels at uninfected conditions.

### XBP-1 splicing assay

The XBP-1 splicing assay was performed essentially as described elsewhere [40]. Briefly, total RNA from the mock or virus (CHIKV/SINV) infected cells was extracted as described above and 1 μg each of the total RNA was used for cDNA synthesis using ImProm II reverse transcription system (Promega), with oligo dT as primer, followed by PCR amplification of XBP-1 spliced genes using XBP-1 splicing specific primers (Materials and Methods). Amplified products were run on 2.5% Agarose gel and visualized under UV ImageQuant.

### Western blotting

HEK293 cells (1×10^5^) were infected with MOI of 1 with CHIKV/SINV and total cell lysate was collected in NET lysis buffer (20 mM Tris, 100 mM NaCl & 1 mM EDTA) containing 0.1% Triton X-100 with protease inhibitor cocktail (Roche) at indicated time points post infections. After 30 min on ice, lysates were centrifuged at 13000 rpm for 10 min and supernatants were used to quantitate the amount of total protein by BCA assay (Pierce). Equal amount (2-5 μg each) of protein was loaded on 12% SDS PAGE followed by Western blotting. Blots were blocked overnight with blocking solution [2% Fish gelatin (sigma) in 1X PBS] and were probed using primary antibodies against various proteins: GFP (Abcam), BIP (Abcam), ATF-6 (Abcam), HSP-90 (cell signaling), p58IPK (cell signaling), CHOP (cell signaling), phospho (Thr 980) PERK (cell signaling), eIF2α (cell signaling) and phospho (Ser 51) eIF2α (cell signaling). Anti-GAPDH antibody (cell signaling) and anti-Actin antibody (sigma) were used as the loading control antibodies. All the antibodies used were diluted in blocking solution. After incubating with secondary HRP-conjugated antibodies, blots were developed using ECL detection reagent (GE healthcare) and exposed on Amersham hyper films prior to development or visualized using Image-quant chemiluminiscent machine. Where required, image quantification was done using Image-J software.

### Construction of CHIKV-pEGFP clones

Vector pEGFP-C1 (Clontech) was used to clone all the four non-structural (nsP1-4) and three major structural (C, E2 & E1) genes of CHIKV. Briefly, CHIKV RNA was extracted using a viral RNA extraction kit (Qiagen). All the genes were amplified using gene specific primers (Materials and Methods) and superscript III one step RT PCR with platinum Taq kit (Invitrogen) in a thermal cycler (Applied Biosystem). Amplified genes were run on 1% agarose gel and amplicons were gel eluted using QIA-quick gel extraction kit (Qiagen). Individual purified PCR products were then inserted in to the pEGFP-C1 vector using cloneEZ PCR cloning kit (Genscript) as per the manufacturer’s recommendations. For convenience of restriction digestion analysis for screening positive clones, nsP1 was inserted in between HindIII-PstI restriction sites and nsP2-4 and C were cloned using XhoI-KpnI restriction sites. Similarly, E1 and E2 were cloned using HindIII-BamHI restriction sites. All the positive clones were further confirmed by DNA sequencing.

### Transfection of plasmids

For transfection of plasmid DNA into HEK293 or MRC-5 cells, cells were seeded to 70% confluency in a 24 well plate (Nunc) and incubated overnight in 37°C incubator supplemented with 5% CO_2_ atmosphere. One μg of each of the plasmids (GFP vector, GFP-nsP1/2/3/4 or GFP-C/E1/E2) was transfected using jet prime transfection reagent (Polypus BST scientific) as per the manufacturers described protocol. Transfected cells were incubated for 48h for protein expression and then washed once with 1X-PBS (Gibco). Finally, cells were collected in TNET-lysis buffer as described above and then subjected to Western blotting. The transfection efficiencies by fluorescence microscopic visualization for each of the plasmids except GFP-nsp2 were measured to be around ~70% using polyplus jet prime transfection reagent, strictly as per the manufacturer’s protocol. For GFP-nsP2 transfection was done using 2 μg of the plasmid and nearly 60% of transfection efficiency was achieved. No cytotoxicity was observed upon transfection of plasmids till 72h post transfection. However, with GFP-nsP2 some cytotoxicity (less than 20% cell death) was observed after 48h post transfection.

### Immunofluorescence

HEK293 cells were seeded on coverslips at a density of 1×10^5^ cells/well in a 12-well plate. Following incubation for overnight at 37°C with 5% CO_2_, the cells were infected with CHIKV or SINV at an MOI of 1. At indicated time points after infection cells were fixed with ice cold 80% acetone for 10 min followed by overnight incubation with blocking buffer (5% BSA in 1X PBS) at 4°C. The CHIKV RNA was detected using monoclonal dsRNA antibody (J2). The phosphorylated form of ER resident protein eIF2α was detected using antibody against phospho (Ser 51) eIF2α (cell signaling). Secondary antibodies used were anti-mouse alexa 488 and anti-rabbit alexa 594. All the antibodies used were diluted in blocking buffer. The coverslips were mounted on glass slides using prolong gold anti-fade mounting medium (Invitrogen) containing DAPI. Immunofluorescence images were captured using an inverted fluorescence microscope (Olympus IX71, USA) or upright confocal microscope (Zeiss) and image analysis was performed with Image-J software.

### Statistics

Statistical comparison of results were performed using unpaired Student’s t test on the GraphPad Prism 5.0 software with p<0.005 considered statistically significant.

## Results

### Growth kinetics of CHIKV and SINV *in vitro*

**Figure 1:**
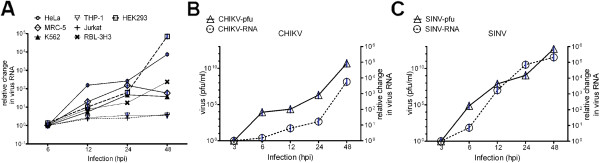
**Growth kinetics of CHIKV and SINV in HEK293 cells. A**) Real time RT-PCR analysis shows the growth kinetics of CHIKV in various cultured mammalian cell types and are presented as fold change in virus RNA (CHIKV nsP1 gene) over 6h infection time point using nsP1 specific primers (Materials and Methods) after normalization with Actin and GAPDH mRNA. The graph is representative of two independent repeats. **B, C**) HEK293 cells (1×10^5^ cells) were infected with MOI-1 of CHIKV or SINV. At indicated time points post infection, infectious virus particles were quantified in the supernatant using standard plaque assay method (left Y-axis in the graph) and real time viral RNA quantification was done on the total RNA extracted from infected cells using specific primers (Materials and Methods) against nsP1 in CHIKV or E1 in SINV. Viral RNA is presented as fold change over 3h infection time points after normalization with Actin and GAPDH mRNA (right Y-axis in the graph). The graphs are representative of three independent repeats.

Since the study is primarily investigating CHIKV growth, we first determined the infectivity and growth kinetics of CHIKV in various cultured mammalian cell types in order to align our data with others in the field. Virus infection was achieved using MOI of 1 and at various time points post infection, growth kinetics was measured using standard plaque assay or by real time RT PCR for viral RNA detection. Mammalian mesenchymal cell types such as human lung fibroblast cells (MRC-5), human cervical epithelial cells (HeLa), human embryonic kidney cells (HEK293) and rat basophilic mast cell like cells (RBL-2H3) support prolific CHIKV replication reaching viral RNA induction up to 10^4^ fold in the infected cells (Figure 1A, B). However, several key immune cells like primary human peripheral blood mononuclear cells (PBMC) (data not shown), peripheral blood monocytic cells (THP-1 & K562) and T lymphocytic cells (Jurkat) were found to be poorly infected with CHIKV, suggesting that immune cells may not be the primary targets for infection (Figure 1A). These findings are in agreement with previous reports that immune cells, including monocyte-derived macrophages and T and B cells are poorly susceptible to CHIKV infection [41,42]. Human embryonic kidney cells (HEK293) are widely used in the study of molecular pathways as they are robust with respect to transfection of foreign genes or proteins [16,43,44]. Indeed HEK293 cells supported CHIKV replication with plaque titers reaching ~10^11^ pfu/ml and up to 10,000-fold induction of viral RNA (Figure 1B). Equally, SINV growth in HEK293 cells under similar conditions was also robust with plaque assay titers of ~10^12^ pfu/ml and nearly 100,000-fold induction of viral RNA (Figure 1C). These high viral titers were also observed in other publications [45,46]. The similarity in growth kinetics of CHIKV & SINV in HEK293 cells made this a relevant model for further investigation into the mechanism by which these viruses modulate the cellular UPR pathway to achieve the high viral load that is often observed in patients [3,12,47].

### The ATF-6 signaling branch of UPR pathway during CHIKV and SINV infection

**Figure 2:**
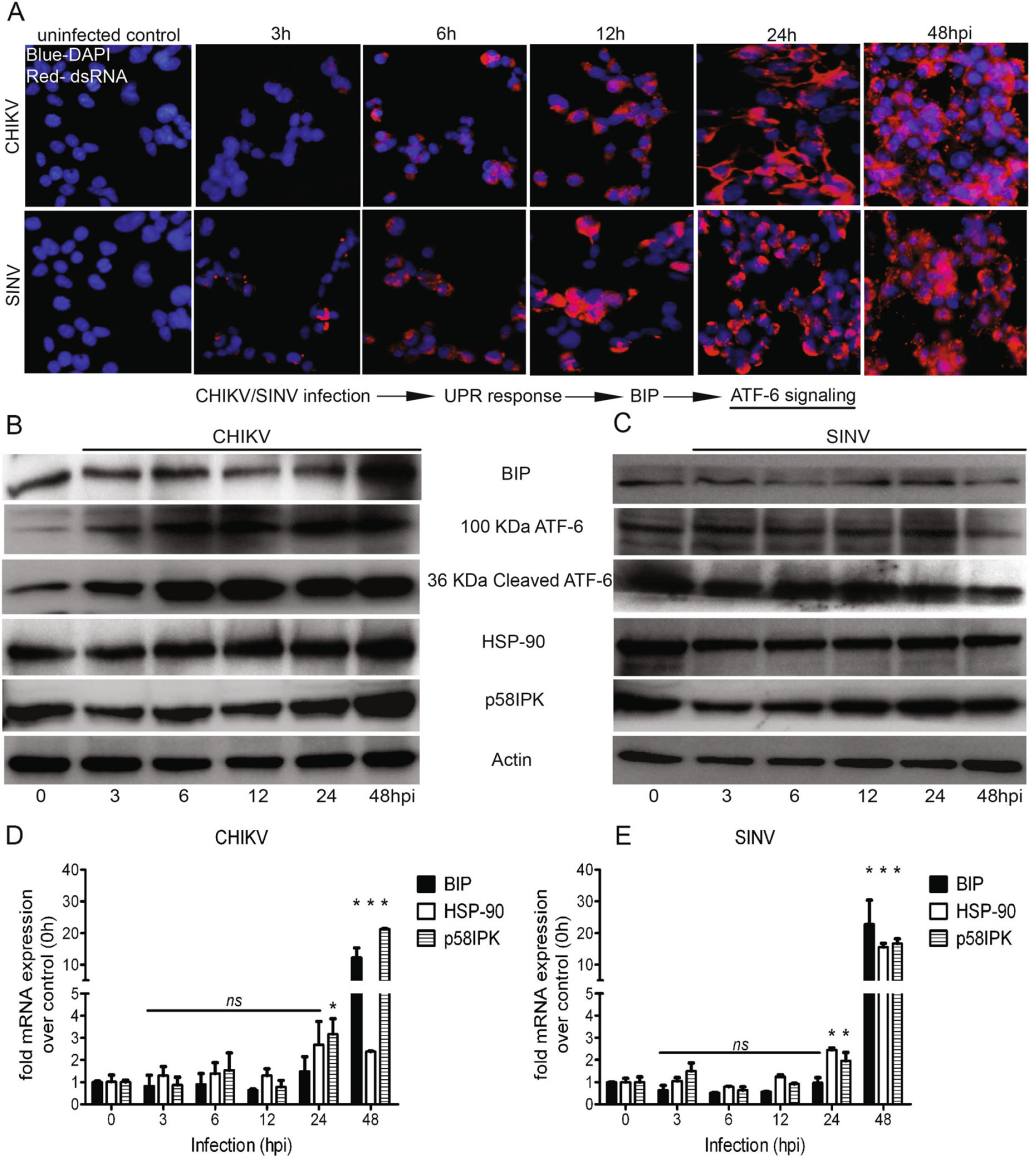
**The ATF-6 signaling during CHIKV and SINV infection. A**) HEK293 cells (1×10^5^ cells) were cultured on coverslips and either mock or CHIKV/SINV infected at an MOI-1. At indicated time points post infection cells were fixed and immunofluorescence microscopy was performed to probe the virus replication-using antibody against dsRNA (red). Uninfected cells were used as negative control and nuclear stain DAPI (blue) was used as background control. From 12h post infection onwards ~90% of cells were infected with CHIKV/SINV and stained positive for dsRNA antibody. **B, C**) HEK293 cells (1×10^5^ cells) were infected with MOI-1 of CHIKV/SINV and at indicated time points post infection cells were lysed using TNET lysis buffer. Lysed samples were run on 12% SDS PAGE followed by Western blotting. Antibodies against BIP, ATF-6, HSP-90 and p58IPK were used to probe the ATF-6 pathway component protein levels during CHIKV/SINV infection. Anti-actin antibody was used to probe loading control and uninfected cells (0h) were used as baseline protein level control. **D, E**) Under the similar experimental conditions and time points stated above, real time RT PCR analysis of BIP, HSP-90 and p58IPK transcripts was done on total RNA extracted from CHIKV/SINV infected cells using specific primers (Materials and Methods) against each of three genes. All three transcripts are presented as fold change over 0h (uninfected cells) after normalization with Actin and GAPDH mRNA. The graphs were plot using graph-pad prism software and are representative of three independent repeats. Any significant change over 0h was determined using student unpaired T test and considered significant (*) if p value was less than 0.05.

Overload of viral protein translation in the ER during virus replication triggers the activation of the UPR pathways. We sought to investigate both the overall and specific impact of CHIKV and SINV replication on the UPR pathway by dissecting the individual signature branches of UPR: the ATF-6, IRE-1 and PERK. For this, HEK293 cells were infected with CHIKV or SINV at an MOI of 1 and at indicated time points (0, 3, 6, 12, 24 and 48 h) post infection, cells were harvested, lysed and subjected to protein and RNA analysis for the component genes of ATF-6 pathway. We first confirmed by using immunofluorescence microscopy that majority of the cells were infected from 12 h post infection onwards, with >95% staining positive for dsRNA for both CHIKV and SINV infections from 24 h post infection (Figure 2A). In response to ER stress BIP activates ATF-6 to auto-proteolyse and induce the transcription of ER chaperone genes such as BIP, HSP-90 and p58IPK [48,49]. During CHIKV infection BIP was induced both at the transcriptional (~12 fold) and translational level (~6 fold) at 48 h post infection (Figure 2B, D). The protein levels of both trans-membrane and cleaved cytosolic ATF-6 were increased throughout the infection time course compared to the uninfected control (0 h) (Figure 2B). The protein levels of ER chaperones, HSP-90 (~2 fold) and p58IPK (~1.5 fold) were also induced from 12 h post infection (Figure 2B), however, transcription levels were only induced at a statistically significant level (p-value less than 0.05) at 24 h (~2.5 fold) and 48 h (~21fold) time points for p58IPK, and at 48 h (~2 fold) for HSP-90 (Figure 2D). In contrast to CHIKV, during SINV infection, no change in the protein levels of BIP was observed (Figure 2C), however the BIP transcript was significantly induced (~22 fold) at 48 h post infection (Figure 2E). No significant change was observed at the protein levels of both trans-membrane and cytosolic cleaved ATF-6 (Figure 2C). Also the protein levels of both HSP-90 and p58IPK were not significantly altered (Figure 2C). However, statistically significant induction of the transcripts for p58IPK (2, 16 fold) and HSP-90 (2.5, 16 fold) were observed at 24 and 48 h post infection (Figure 2E). Taken together, the data here suggest that the ATF-6 pathway signaling is significantly activated during CHIKV infection, whereas the SINV infection appears to not have a major modulatory effect on this branch of the UPR pathway.

### The IRE-1 signaling branch of UPR pathway during CHIKV and SINV infection

**Figure 3:**
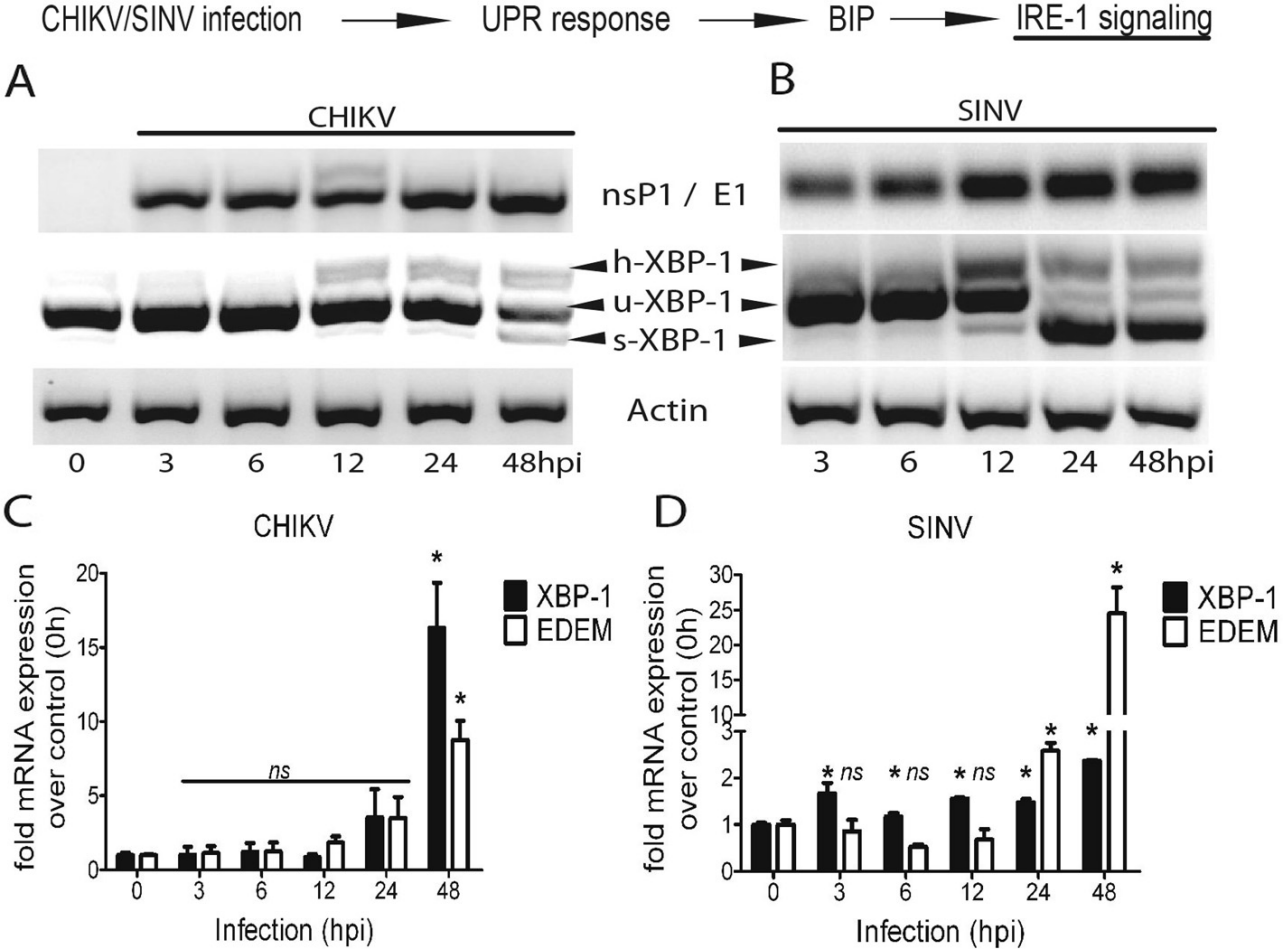
**The IRE-1 signaling during CHIKV and SINV infection. A, B**) HEK293 cells (1×10^5^ cells) were infected with MOI-1 of CHIKV/SINV and at indicated time points post infection total RNA was extracted to make the cDNA. Equal amounts (1μg each) of cDNA were used for PCR based XBP-1 splicing assay using specific primers against the spliced (s-XBP-1) and un-spliced (u-XBP-1) gene variants. Virus replications in the same samples (CHIKV/SINV) are probed using nsP1 specific primer for CHIKV or E1 specific primer for SINV (Materials and Methods). Actin gene amplification was used as an input RNA control and 0h (uninfected cells) was used as baseline control. **C, D**) Under similar experimental conditions and time points stated above, real time RT PCR analysis of XBP-1 and EDEM was done on total RNA extracted from CHIKV/SINV infected cells using specific primers (Materials and Methods) against each of the two genes. Both gene transcripts are presented as fold change over 0h (uninfected cells) after normalization with Actin and GAPDH mRNA. The graphs were plot using graph-pad prism software and are representative of three independent repeats. Any significant change over 0h was determined using student unpaired T test and considered significant (*) if p<0.05.

Next the IRE1 branch was investigated by probing the splicing in the XBP-1 gene, which is a characteristic marker for activation of IRE-1 signaling [50-52]. The spliced XBP-1 gene product acts as transcription factor and activates the transcription of pro-survival genes such as EDEM and BCL-2 family proteins [53,54]. To assess the IRE-1 signaling, upon CHIKV/SINV infections, total RNA was extracted from the infected cells, harvested at various time points post infection and used for cDNA synthesis. The XBP-1 gene-splicing event was detected using a standard primer-based XBP-1 splicing assay [40]. For easier interpretation of data, the corresponding level of viral RNA present at each time point post infection was detected using virus gene specific detection primers for CHIKV (nsP1 gene) and SINV (E1 gene) (Figure 3A, B). The data shows that CHIKV infection triggers moderate XBP-1 splicing from 12 h post infection, which only becomes prominent at 48 h post infection (Figure 3A). Quantitative real time PCR analysis showed that the transcription levels of both XBP-1 gene (~9 fold) and EDEM-1 (~16 fold) increased at 48 h post infection (Figure 3C). However in the case of SINV infection, the spliced XBP-1 gene transcript was much more prominent than was observed for CHIKV, starting from 12 h post infection with dramatic increase in the spliced product at 24 and 48 h post infection (Figure 3B). Real time PCR analysis revealed the increase in transcription of XBP-1 gene starting from 3 h post infection and significant increase in the EDEM transcript at 24 h (~2.5 fold) and 48 h (~24 fold) post infection (Figure 3D). Together the data suggests that both CHIKV and SINV activate the IRE-1 branch of UPR except that SINV infection appears to have a more profound impact on XBP-1 gene splicing from a very early time point.

### The PERK signaling branch of UPR pathway during CHIKV and SINV infection

**Figure 4:**
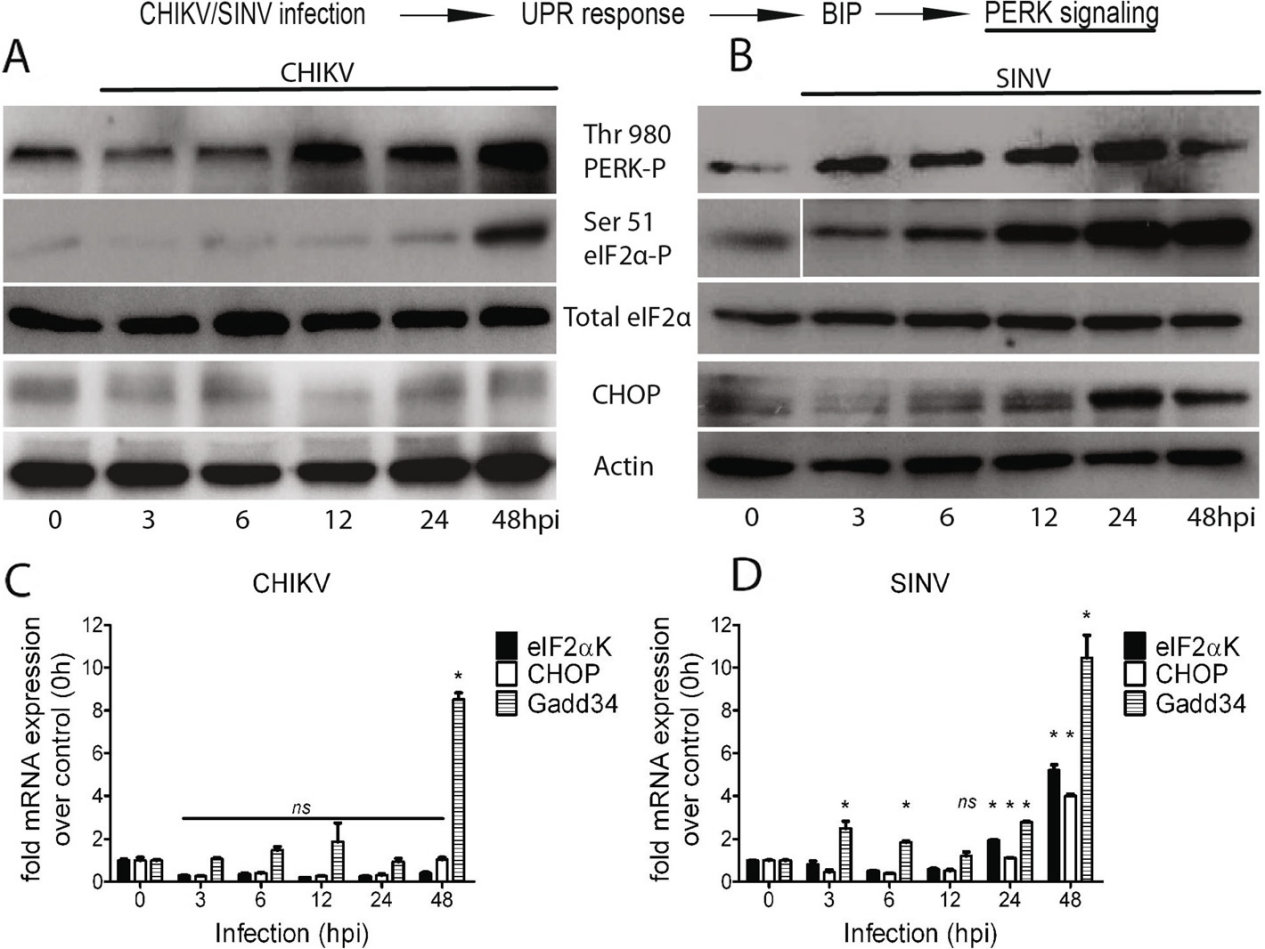
**The PERK signaling during CHIKV and SINV infection. A, B**) HEK293 cells (1×10^5^ cells) were infected with MOI-1 of CHIKV/SINV and at indicated time points post infection cells were lysed using TNET lysis buffer. Lysed samples were run on 12% SDS PAGE followed by Western blotting. Antibodies against phospho (Thr 980) PERK, phospho (Ser 51) eIF2α, eIF2α and CHOP were used to probe the PERK pathway component protein levels during CHIKV/SINV infection. Anti-actin antibody was used to probe loading control and uninfected cells (0h) were used as baseline protein level control. **C, D**) Under similar experimental conditions and time points stated above, real time RT PCR analysis of eIF2αK, CHOP and GADD34 transcripts was done on total RNA extracted from CHIKV/SINV infected cells using specific primers (Materials and Methods) against each of three genes. All three transcripts are presented as fold change over 0h (uninfected cells) after normalization with Actin and GAPDH mRNA. The graphs were plot using graph-pad prism software and are representative of at-least three independent repeats. Any significant change over 0h was determined using student unpaired T test and considered significant (*) if p<0.05.

To examine the effects of CHIKV and SINV replication on the PERK pathway of UPR, antibodies against phsopho (thr 980) PERK and phospho (ser 51) eIF2α were used to measure their respective phosphorylation levels. HEK293 cells were infected with CHIKV or SINV at an MOI of 1 and at 0, 3, 6, 12, 24 and 48h post infection cells were harvested and lysed before being subjected to protein and RNA analysis for PERK pathway component genes. During CHIKV infection the increase in the phosphorylation of PERK was detected starting from 12 h post infection (Figure 4A). Intriguingly, even when the PERK was activated (as indicated by its phosphorylation) no phosphorylation (ser 51) of eIF2α was observed over total eIF2α until 24 h post infection (Figure 4A). However, at 48 h post infection an increase in phosphorylation (ser 51) of eIF2α was observed (Figure 4A) suggesting a delayed cellular response to virus infection and perhaps an implication for the possible role of virus mediated suppression of eIF2α phosphorylation. Similar results were also obtained using another cell type MRC-5 (Additional file 1: Figure S1) thus excluding the possibility that the delayed response is cell-type specific. The transcript level of eIF2αK was not altered during CHIKV infection (Figure 4C). Also, both the protein and transcript levels of downstream apoptosis marker, CHOP, were almost undetectable and not altered at any time points post CHIKV infection (Figure 4A, C**)**. Interestingly, GADD34 a negative regulator of PERK was transcriptionally induced (~9 fold) at 48 h post infection (Figure 4C). However, during SINV infection the PERK signaling was in stark contrast to that observed for CHIKV infection (Figure 4). SINV infection induced phosphorylation of PERK (Figure 4B) and a dramatic increase in the phosphorylation (ser 51) of eIF2α was observed over the entire time course, starting 3h post infection (Figure 4B). Indeed, the transcript levels of eIF2αk were also significantly elevated at 24 (~5 fold) and 48 h (~12 fold) post infection (Figure 4D). CHOP activity was also dramatically increased during SINV infection at both the protein and transcript levels (upto 4 fold) starting 6 h post infection (Figure 4B, D). Overall, the data here suggest that CHIKV may modulate the PERK pathway signaling by suppressing the phosphorylation (ser 51) of eIF2α in the early phase of infection (3-24 h). SINV infection on the other hand leads to an uncontrolled UPR in the cell characterized by increased phosphorylation (ser 51) of eIF2α and apoptosis.

### CHIKV infection suppress phosphorylation (ser 51) of eIF2α

**Figure 5:**
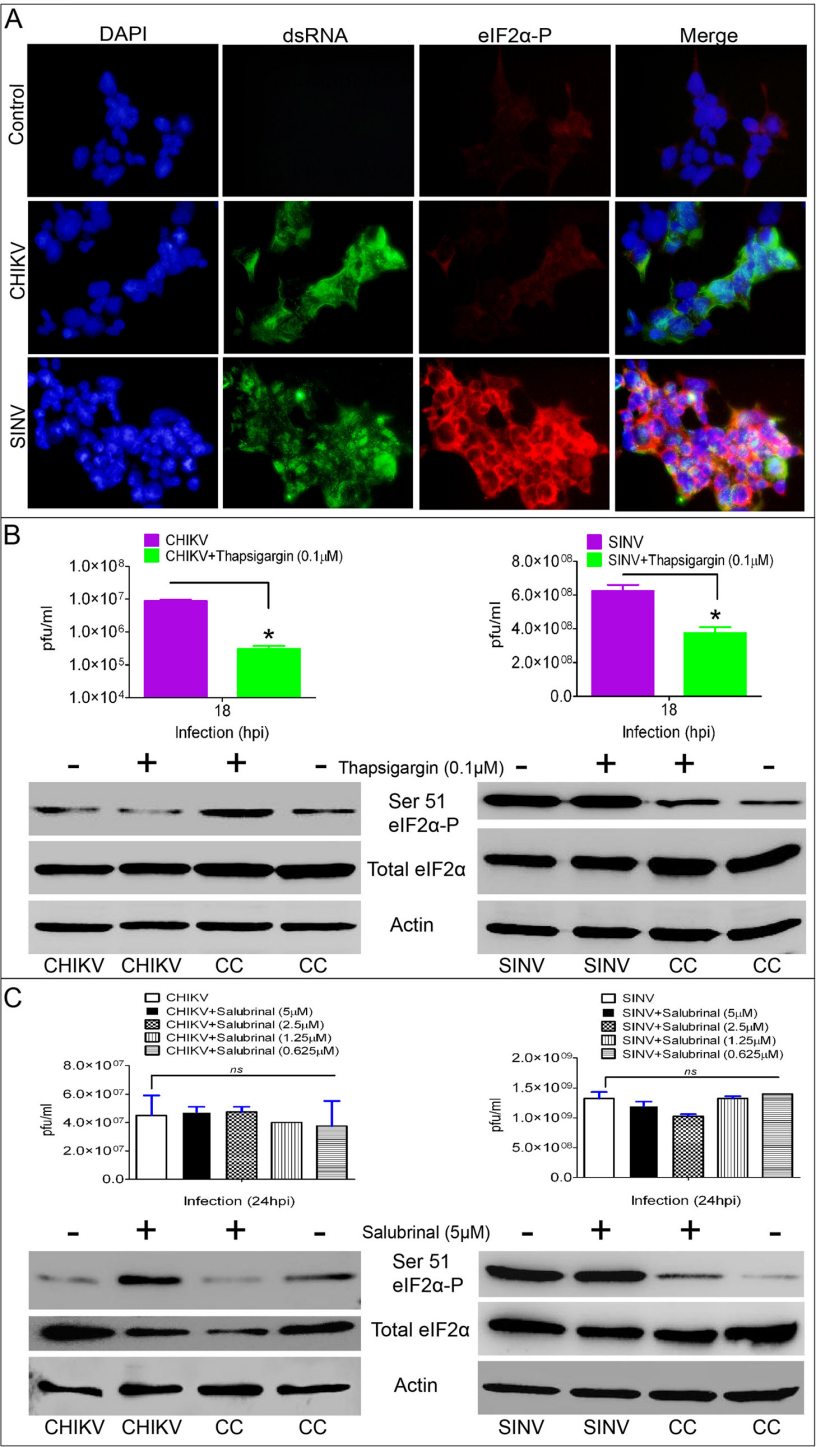
**CHIKV infection suppresses the phosphorylation of eIF2α. A**) Immunofluorescence microscopy at 10X magnification on CHIKV/SINV (MOI-1) or mock-infected HEK293 cells at 24 h. Unlike SINV, CHIKV infection failed to phosphorylate eIF2α (red). Virus infection was probed using dsRNA antibody (green). **B**) HEK293 cells were mock or CHIKV/SINV infected (MOI-1) till 12h to allow the translation of CHIKV encoded proteins followed by treatment with thapsigargin (0.1 μM) for 6h and Western blotting was performed on cell lysates using specific antibodies against phospho (Ser 51) eIF2α and eIF2α. Anti-actin antibody was used to probe loading control and uninfected or untreated cells (CC) were used as baseline control. Plaque assay titers in the presence of thapsigargin for 6h are presented as log pfu/ml. **C**) HEK293 cells were mock or CHIKV/SINV infected (MOI-1) for 1h. Cells were washed twice with 1X PBS to remove any traces of unbound virus particles followed by treatment with medium containing indicated concentrations of salubrinal for 24h. At 24 h media supernatant was used for plaque assay and cells were used for Western blotting using antibodies against phospho (Ser 51) eIF2α and eIF2α. Anti-actin antibody was used to probe loading control and uninfected or untreated cells (CC) were used as baseline control.

To interrogate the delayed phosphorylation (ser 51) of eIF2α during CHIKV infection, we first confirmed by immunofluorescence microscopy that the phosphorylation (ser 51) of eIF2α at 24 h post infection was much more reduced and perhaps even suppressed in comparison to SINV or uninfected controls (Figure 5A). Next, we determined whether CHIKV infection could efficiently suppress phosphorylation (ser 51) of eIF2α even in the presence of thapsigargin or tunicamycin (Figure 5B,Additional file 1: Figure S2A), the known chemical inducers of ER stress [39,55,56]. For this we verified that treatment of HEK293 cells with thapsigargin (0.1 μM) or tunicamycin (0.5 μg/ml) for 6 h induced ER stress resulting in increased protein phosphorylation (ser 51) of eIF2α (Figure 5B, Additional file 1: Figure S2A). Based on this thapsigargin/tunicamycin treatment time of 6 h was selected for further experiments to avoid any undesired toxicity effects of the drug. To examine the effect of CHIKV or SINV replication on thapsigargin/tunicamycin induced ER stress, HEK293 cells were infected with MOI of 1 of CHIKV or SINV for 12 h (for sufficient translation of virus encoded proteins), thoroughly washed twice with FCS free DMEM to remove any traces of excess virus and finally treated with thapsigargin (0.1μM)/ tunicamycin (0.5 μg/ml) or mock treatment for another 6 h. The cells were harvested and lysed for Western blotting analysis and the media supernatants from the tests were used for virus quantification by plaque assay. As expected, the phosphorylation (ser 51) of eIF2α was enhanced (100% eIF2α-P) over total eIF2α in uninfected but thapsigargin or tunicamycin treated cells (Figure 5B, Additional file 1: Figure S2A). At the same time dramatic reduction in the levels of eIF2α (ser 51) phosphorylation (~8% eIF2α-P) over total eIF2α was observed for cells infected only with CHIKV even in the presence of thapsigargin or tunicamycin (Figure 5B, Additional file 1: Figure S2A). However, SINV infection induced massive phosphorylation of eIF2α in both mock and thapsigargin or tunicamycin treated cells (Figure 5B, Additional file 1: Figure S2A**)**. Consistent with our earlier observation (see above in Figure 4, 5A) CHIKV infection by itself (~5% eIF2α-P) failed to phosphorylate (ser 51) eIF2α (Figure 5B). Plaque assay data confirmed the significant reduction in both CHIKV and SINV viral titers upon treatment with thapsigargin for 6h (Figure 5B). Next in order to examine if cellular phosphatases could be directly or indirectly modulating the de-phosphorylation of eIF2α we used ‘salubrinal’ a specific inhibitor of ER phosphatase (PP1c) which function together with GADD34. For this, cells were infected with CHIKV/SINV at an MOI of 1 for 1h followed by treatment with various concentrations of salubrinal starting from 0.625 μM to 5 μM for 24 h. After 24 h post infection and treatment, media supernatant was collected for plaque assay and cells were collected for Western blotting analysis. By plaque assay, salubrinal treatment had no effect on the production of either CHIKV or SINV infectious virus particles. Nevertheless, salubrinal treatment lead to the increased phosphorylation of eIF2α only in CHIKV infected cells suggesting the involvement of GADD34 in CHIKV mediated eIF2α de-phosphorylation (Figure 5C). In SINV infection salubrinal treatment had no significant increase in the phosphorylation of eIF2α over untreated infected cells (Figure 5C).

### CHIKV protein nsP4 suppresses phosphorylation (Ser 51) of eIF2α

**Figure 6:**
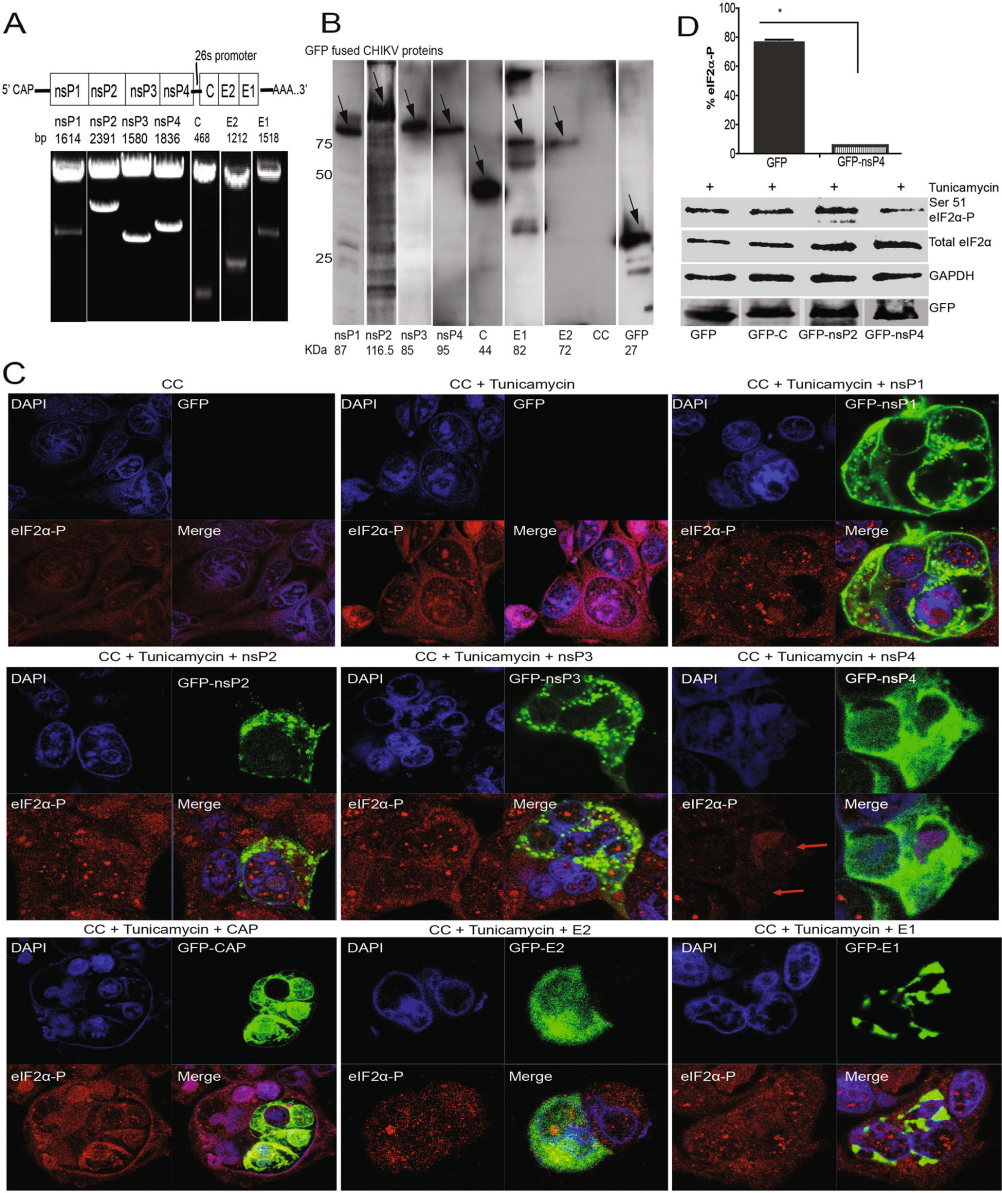
**CHIKV nsP4 suppresses the phosphorylation of eIF2α. A**) Schematic diagram showing the organization of CHIKV genes in the viral genome. CHIKV genes (nsP1-4, C, E1 &E2) were cloned in a CMV driven GFP tagged vector and restriction analysis in Figure 6A shows expected size insert in respective positive clones. **B**) HEK293 cells were transfected with 1 μg each of the recombinant CHIKV-gene plasmids and incubated for 24 h to permit protein translation. At 24 h, Western blotting was performed with anti-GFP antibody for the detection of the correct size proteins. Cells treated with transfection reagent without plasmid were used as negative control (CC). **C**) HEK293 cells were transfected with 1μg each of CHIKV-gene carrying plasmids or control GFP-gene plasmid for 24h and then treated with tunicamycin (0.5 μg/ml) for 24 h followed by immunofluorescence detection using antibody against phospho (Ser 51) eIF2α (red) under the confocal microscope and images were captured at 63X magnification. Nuclear stain DAPI (blue) was used to differentiate single cells and individually expressed CHIKV proteins were visualized as GFP-fused proteins (green). As indicated using red arrows staining of phospho (Ser 51) eIF2α (red) was dramatically reduced in cells expressing CHIKV nsP4. Images were processed using Zen image software from Zeiss. **D**) Cells (HEK293) were transfected with C, nsP2 and nsP4 or a control plasmid GFP for 24 h and then further treated with tunicamycin (0.5μg/ml) for 24 h followed by Western blotting using antibodies against phospho (Ser 51) eIF2α, and eIF2α. Anti-GAPDH was used as loading control and anti-GFP was used to probe plasmid encoded protein expressions. Change in band intensity of phospho (Ser 51) eIF2α in GFP and nsP4 transfected cells was calculated using image-J and presented as % eIF2α-P over total eIF2α. The data is representative of two independent experiments. The symbol (*) denotes p < 0.05.

To understand mechanism by which CHIKV replication suppresses eIF2α (Ser 51) phosphorylation and also to explore the possibility of whether any of the CHIKV-encoded proteins could play a role in this process, we individually cloned all the major structural and non structural genes (Figure 6A) into a CMV promoter driven GFP tagged vector. The primers listed in Materials and Methods were used to amplify the CHIKV genes from the cDNA obtained from viral RNA and the resulting correct size fragments were cloned into pEGFP-C1 vector by recombination cloning as described in the Materials and Methods section. The sequence verified clones (1 μg of each of the plasmid) were used to transfect HEK293 cells followed by incubation for 24 h to allow sufficient translation of plasmid-encoded proteins. SDS PAGE separation followed by Western blotting using anti-GFP antibody confirmed that GFP-fused CHIKV proteins were expressed and each migrated to the correct size (Figure 6B). In the case of GFP-E1 expression, three other bands were observed in addition to the expected size of 87 KDa (Figure 6B). We speculate that being a surface glycoprotein, the higher band could be a multimeric form of GFP-E1, while the lower bands may be due to degradation product. To address the question whether any of these individually transfected CHIKV genes could suppress tunicamycin-induced eIF2α (Ser 51) phosphorylation we transfected the individual GFP-fused CHIKV genes in HEK293 cells followed by an incubation period of 24 h to allow the sufficient translation of cloned genes. This was followed by tunicamycin (0.5 μg/ml) treatment and further incubation for 24h prior to fixing and visualizing using confocal immunofluorescence microscopy or harvesting cells and analysis by Western blotting. Remarkably, of the eight CHIKV gene constructs that were transfected, only the expression of CHIKV nsp4, which is the RNA-dependent-RNA polymerase, efficiently suppressed the phosphorylation (Ser 51) of eIF2α, even in the presence of tunicamycin (Figure 6C, D). However, other CHIKV proteins such as nsP1, nsP2, nsP3, C, E2, E1 and the control protein GFP had no effect on the phosphorylation (Ser 51) of eIF2α (Figure 6C, D and other representative data which is not shown here. In order to negate the possibility that the nsP4 mediated suppression of the phosphorylation (Ser 51) of eIF2α may be due to a cell-line artifact; CHIKV-GFP constructs were also examined in MRC-5 fibroblast cell line. The results showed the similar trend of suppression of eIF2α phosphorylation when MRC-5 cells were transfected with CHIKV nsP4 (Additional file 1: Figure S2B). Cumulatively, our data suggest that expression of CHIKV nsP4 significantly reduces the phosphorylation (Ser 51) of eIF2α thus ensuring translation of viral proteins.

## Discussion

Virus infection in mammalian cells consists of a series of events from entry to maturation and egress of virus. Remarkably, as intracellular parasites, viruses rely on the utilization of cellular machinery and resources to complete their life cycle. In this complex process, RNA viruses synthesize dsRNA intermediates and produce viral proteins within host cells. Consequently, viral replication elicits cellular responses, such as ER stress and the interferon response, as a first line of defense against the invading pathogen. To overcome this natural resistance, viruses have evolved various mechanisms to subvert host responses that limit or inhibit viral replication. Recently, several groups [44,57-59] have reported the impact of CHIKV or SINV replication on host cellular interferon and apoptotic machinery. In this study we specifically examined the cellular UPR signaling during CHIKV and SINV infections and show that the gene/protein expression responses in the pathway are differentially modulated although the two viruses are considered to be closely related to each other. We explored in more detail the mechanistic basis for CHIKV modulation of the UPR pathway.

The stimulation of transcription and translation of BIP (the master regulator of UPR) has been observed for several viruses [33,60]. Not surprisingly the massive replication of CHIKV resulted in the induction of ER resident chaperones, such as BIP and HSP-90, which presumably assists in the folding of unfolded proteins in order to relieve the UPR stress within the cell. SINV infection, on the other hand, did not show significant induction in the expression of BIP and HSP-90, suggesting the possible early buildup of ER stress, which may contribute to the apoptosis and early cell death that was observed [61]. However SINV infection caused a more pronounced IRE-1 mediated splicing of XBP-1 gene that resulted in transcriptional induction of XBP-1 and EDEM, a pro-survival gene-product. Although the induction of XBP-1 and EDEM was less prominent during CHIKV infection in comparison to SINV infection, the present data is consistent with the recently reported role of IRE-1 signaling in delaying caspase-induced cell death [62]. In the PERK branch of UPR pathway, the phosphorylation of PERK was observed in both CHIKV and SINV infected cells but intriguingly the kinetics of the concomitant phosphorylation of eIF2α showed marked difference between the two. At the early time points following CHIKV infection although increased PERK phosphorylation could be detected from 12 h post infection, the phosphorylation of eIF2α was not detected until 48h post infection whereas in SINV infected cells the eIF2α phosphorylation could be detected from 3 h post infection. This discrepancy was addressed by treating CHIKV infected cells with thapsigargin or tunicamycin, the well known strong inducers of PERK and eIF2α phosphorylation. This clearly demonstrated that eIF2α phosphorylation in the cell was suppressed at the early stages of CHIKV infection (3-24 h) even with thapsigargin or tunicamycin treatment so as to allow high and sustained viral protein production without building up the ER stress. At 48 h post CHIKV infection the eIF2α phosphorylation was quite prominent and comparable to the level observed at the same time point in SINV infected cells. However at this time point GADD34, a negative regulator of PERK, which mediates the de-phosphorylation of phospho-eIF2α and p58IPK, a chaperone, which suppresses the PERK mediated phosphorylation of eIF2α were also induced, suggesting that even when the cell tries to overcome its control by CHIKV infection, negative loop transcripts like GADD34 and p58IPK are activated in order to rescue viral protein synthesis. To further explore the importance of GADD34 in mediating CHIKV induced suppression of eIF2α-phosphorylation we used a specific GADD34 inhibitor ‘salubrinal’. Interestingly salubrinal treatment during CHIKV infection lead to an increased phosphorylation of eIF2α suggesting the involvement of GADD34 in suppression of eIF2α-phosphorylation. Salubrinal treatment during SINV infection however did not show any significant change in the phosphorylation of eIF2α over untreated SINV infected cells. Also, interestingly CHOP activity was not detected at both protein and transcription levels throughout the CHIKV infection time course. In stark contrast to CHIKV, SINV infection leads to phosphorylation of PERK and a dramatic increase in the phosphorylation of eIF2α starting from 3h post infection. The enhanced expression of CHOP detected as early as 3h suggests the signature cell death by apoptosis during SINV infection. Although, GADD34 was transcriptionally induced during SINV infection the heightened phosphorylation of eIF2α and further increase in CHOP activity triggers massive cell death, which could be observed starting from 12 h post infection (data not shown). Altogether, our data suggest that the PERK branch of UPR pathway is regulated during CHIKV infection as reflected by the suppression in the phosphorylation of eIF2α during the early stage of infection and the reduced CHOP activity.

A mechanistic basis for the suppression in the phosphorylation of eIF2α during the early stage of CHIKV infection was investigated using EGFP-tagged clones of seven CHIKV proteins and we discovered that the observed phenotype in the PERK pathway (i.e. suppression of the phosphorylation of eIF2α) is mediated by CHIKV nsP4 protein, which contains the RNA-dependent-RNA polymerase activity. An interesting conjunction to our finding is that nsP4 protein of alphavirus is the first non-structural protein to be cleaved from the nsP1-4 polyprotein. and this cleavage as well as its enzymatic activity play a critical role in the synthesis of minus strand viral RNA [4]. Furthermore it is also well known that the alphavirus nsP4 is unstable, short-lived and degrades rapidly in the infected cell [63]. This instability of nsP4 could possibly explain why infected cells recover some degree of eIF2α phosphorylation in the late phase of infection (48 h). Together, we suspect that early suppression of the translation inhibition involving nsP4 could permit the buildup of template RNA for further translation and, thereby, support robust replication.

The question of how CHIKV regulates the host translational machinery to achieve a high level of replication is important to examine in detail particularly in light of seemingly contradictory reports on this topic. White et al. [59], reported independence of CHIKV induced translational shut-off from the phosphorylation of eIF2α, an intriguing finding since eIF2α phosphorylation has a well established role in the shut-off of the host translational machinery [64]. However, in our detailed time course experiments with HEK293 cells, we did not observe eIF2α phosphorylation until 48 h post infection, which was also consistently not observed in another cell type MRC-5 cells until 48 h. We believe our detailed time course study provides advantage in understanding the complex early events of virus-host interactions in the UPR pathways. That it occurs, mechanistically, is interesting since the actions of transiently stable nsP4 function correlate to viral RNA replication and life cycle. Even at the late phase of infection induction of ER chaperones (BIP, HSP-90) along with pro-survival gene-product (EDEM) could work synergistically with negative regulators of eIF2α phosphorylation (p58IPK, GADD34) to possibly support sustained CHIKV replication. SINV infection, on the contrary, is characterized by uncontrolled UPR as reflected by its failure to induce synthesis of ER chaperones followed by increased phosphorylation of eIF2α and CHOP activity leading to early cell death. Since both CHIKV and SINV infections showed differential activation or modulation of the UPR, further detailed studies on the effects of infection on host cellular UPR machinery is required to better understand their characteristic prolific replication profiles.

In conclusion, we show that the two closely linked viruses CHIKV and SINV from the same family, responds differently to the host cellular UPR machinery. Indeed, CHIKV infection modulates the PERK branch of UPR machinery and that it occurs mechanistically through the involvement of the viral protein nsP4 in direct or indirect conjunction with host factors such as GADD34. The early suppression of UPR provides a mechanism for robust replication. Our observation opens up the possibility to explore in detail the interplay of CHIKV nsP4 protein in establishing the infection and exploit possible avenues to use this in identifying a suitable target for antiviral intervention.

## Competing interests

The authors declares that they have no competing interests.

## Authors’ contributions

AR carried out all the experiments and analysis reported in this manuscript. SV conceived the study. SV and MN participated in design and analysis of experiments together with AR and helped to draft the manuscript. All authors read and approved the final manuscript.

## Supplementary Material

Additional file 1 Supplementary_Material_Rathore et al. Click here for file
