# Supplementary material for: Differential unfolded protein response during Chikungunya and Sindbis virus infection: CHIKV nsP4 suppresses eIF2α phosphorylation
Source: Virol J. 2013 Jan 28;10:36. doi: 10.1186/1743-422X-10-36 (PMC3605262; doi:10.1186/1743-422X-10-36)

**Supplementary figure legends**

**Fig.S1**

**A)** MRC-5 cells (1×10^5^ cells) were infected with MOI-1 of CHIKV/SINV and at indicated time points post infection cells were prepared for immunofluorescence microscopy as described in Materials and Methods using antibodies against dsRNA and DAPI and imaging was done using inverted fluorescence microscope at 10X magnification.

B) MRC-5 cells (1×10^5^ cells) were infected with MOI-1 of CHIKV/SINV and at indicated time points post infection cells were lysed using TNET lysis buffer. Lysed samples were run on 12% SDS PAGE followed by western blotting. Antibodies against phospho-PERK (Thr 980), phospho (Ser 51) eIF2α and eIF2α was used to probe phosphorylated PERK, eIF2α and total eIF2α. Anti-actin antibody was used to probe loading control and uninfected cells (0h) were used as baseline protein level control.

**Fig.S2**

**A)** HEK293 cells were mock or CHIKV/SINV infected (MOI-1) till 12h to allow the translation of CHIKV encoded proteins followed by treatment with tunicamycin (0.5μg/ml) for 6h and Western blotting was performed on cell lysates using specific antibodies against phospho (Ser 51) eIF2α and eIF2α. Anti-actin antibody or GAPDH antibody was used to probe loading control and uninfected or untreated cells (CC) were used as baseline control.

**B)** HEK293 or MRC-5 cells (1x10^5^) were transfected with GFP fused nsP1, nsP2, nsP4, E1 and E2 and GFP vector for 24h and then further treated with tunicamycin (0.5μg/ml) for 24h followed by western analysis using antibodies against eIF2α-P, eIF2α and GFP. Anti-actin or GAPDH was used to probe loading control. Change in band intensities were calculated using image-J and presented as % eIF2α-P over total eIF2α.


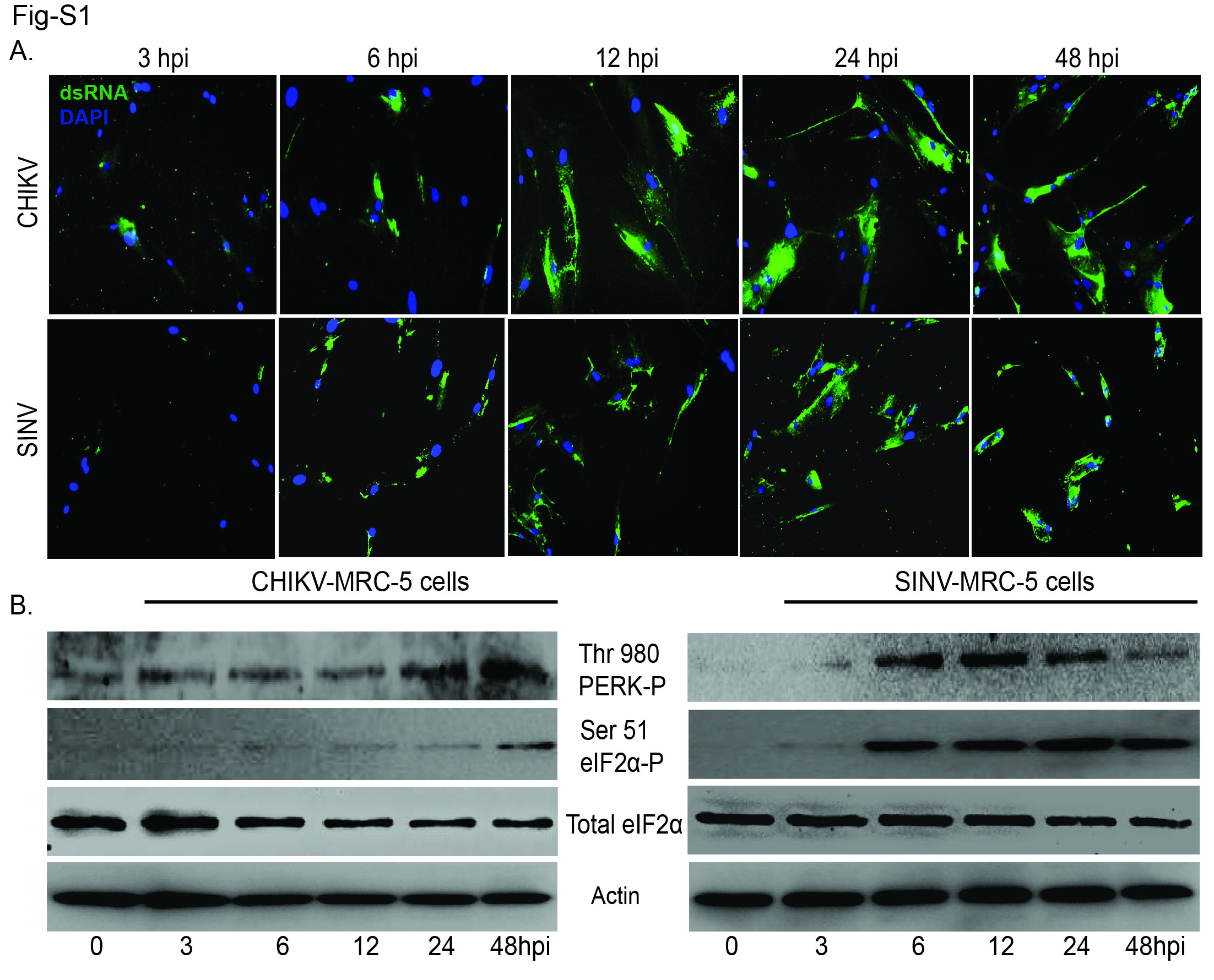


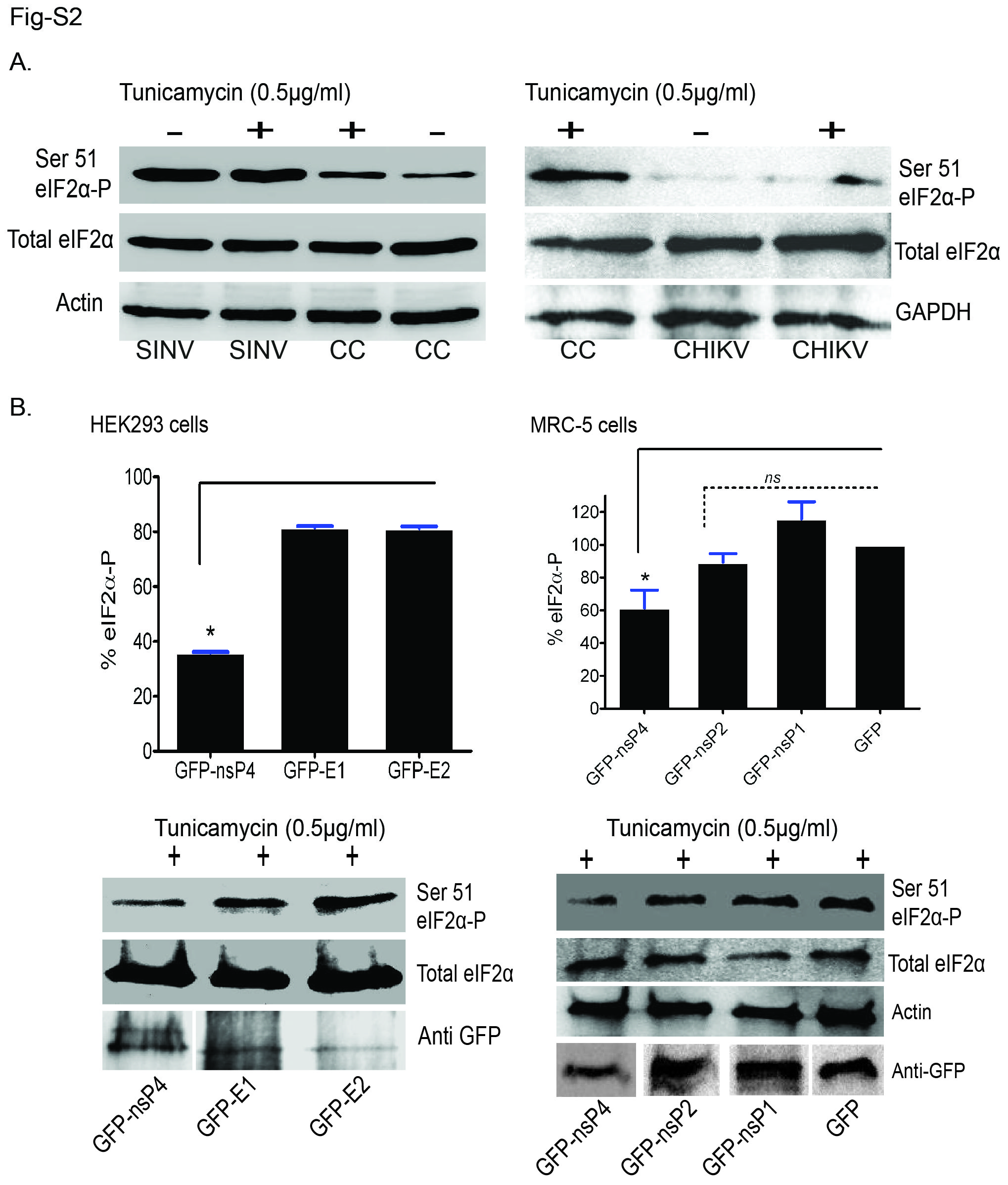

Supplement: Additional file 1 — Supplementary_Material_Rathore et al. [file 1743-422X-10-36-S1.docx]
